# Diversity of adenosine deaminase in children with EBV-related diseases

**DOI:** 10.1186/s13052-022-01338-y

**Published:** 2022-08-19

**Authors:** Ting Shi, Yu Shen, Wei Zhang, Meiying Qian, Xiuli Chen, Linlin Huang, Jianmei Tian

**Affiliations:** 1 Department of Infectious Diseases, Soochow University Affiliated Children’s Hospital China, 303 Jingde Road, Suzhou, Jiangsu China; 2 Jiangsu Institute of Clinical Immunology, The First Affiliated Hospital of Soochow University, Suzhou, China

**Keywords:** Adenosine deaminase, Epstein-Barr virus, Viral load

## Abstract

**Background:**

Adenosine deaminase (ADA) is an enzyme involved in purine metabolism with an important role in cellular immunity. Thus, this study investigated the association between ADA and Epstein–Barr virus (EBV)-related diseases.

**Methods:**

We retrospectively collected data from all children admitted to the Children’s Hospital of Soochow University, Suzhou, China, between May 1, 2018, and March 31, 2019, who underwent plasma EBV-DNA polymerase chain reaction, alanine aminotransferase (ALT), and ADA testing.

**Results:**

Of 6868 children, 1877 had an elevated level of ADA, and 4991 had a level within the normal range. Multivariate logistic regression analysis indicated that ALT (adjusted odds radio [aOR] = 1.001, 95% confidence interval [CI]: 1.001–1.002), EBV infection (aOR = 8.486, 95% CI: 6.753–10.663), inflammatory disease (aOR = 3.915, 95% CI: 3.198–4.794), autoimmune disease (aOR = 2.307, 95% CI: 1.823–2.920), and malignant disease (aOR = 1.381; 95% CI: 1.101–1.734) were risk factors for an elevated ADA level. Furthermore, the ADA levels among EBV-related diseases significantly differed, including infectious mononucleosis, atypical EBV infection, respiratory infection, malignant disease, and other diseases (*P* < 0.05). In addition, the ADA level positively correlated with the Epstein–Barr viral load (r = 0.501, *P* < 0.05).

**Conclusions:**

This large, retrospective study identified a correlation between ADA and EBV-related diseases, which may help clinicians detect these diseases earlier based on the plasma ADA concentration.

## Background

Adenosine deaminase (ADA) is a multifunctional protein closely related to purine metabolism and immune function. Previous studies have confirmed ADA’s pivotal role in the growth and differentiation of lymphocytes, natural killer cells, and macrophages; it is also a marker of T lymphocyte-mediated cellular immunity [1]. Furthermore, ADA is closely associated with inflammation, autoimmune diseases, acquired immune deficiency syndrome, and tumors, with its concentration positively correlating with disease severity [2–4]. Numerous studies have demonstrated that injury, necrosis, and increased membrane permeability of hepatocytes increase serum ADA activity in hepatitis B, hepatitis C, and autoimmune hepatitis [5]. Mejer et al. [6] also found that patients with infectious mononucleosis (IM) had an increased level of ADA in a small-sample study, but associations between the ADA level and other Epstein–Barr virus (EBV)-related diseases were not described.

EBV is a gamma herpes virus infecting at least 95% of adults worldwide [7]. In developing countries, EBV exposure mainly occurs in children around the age of 5 years. In developed countries, infection onset is delayed, but adults are almost uniformly positive [8]. Most infections in younger children are benign and often subclinical [7]. The most common acute presentation of EBV infection is febrile viral upper respiratory illness [7], and young adults are more likely to present with IM [9]. However, some children with EBV infection develop hemophagocytic syndrome or progress to chronic active EBV infection. Additionally, EBV is associated with autoimmunity and tumors [10, 11]. EBV infection can also activate cytotoxic T-lymphocytes (CTLs) and natural killer cells, and activated CTLs causes a cell-mediated immune response, inducing the clinical presentation of primary infection [11].

ADA’s involvement in the EBV-mediated immune response remains unclear. Therefore, this study investigated the risk factors for an abnormally high plasma ADA concentration, compared the plasma ADA concentrations among EBV-related diseases, and explored the correlation between the ADA level and the EBV viral load. We aimed to determine the feasibility of using ADA as a diagnostic or disease severity marker for EBV infection.

## Methods

### Data sources and study design

We performed a retrospective, observational study at a tertiary care hospital (Children’s Hospital of Soochow University) in Suzhou, southeastern China, from May 1, 2018, to March 31, 2019. We included all inpatients who underwent plasma Epstein–Barr (EB) nucleic acid, EBV-specific antibodies, and liver function assays. We collected data on patient demographics, plasma EBV DNA, EBV-specific antibodies, alanine aminotransferase (ALT) and ADA levels at admission, and diagnostic information (based on the discharge diagnosis).

We enrolled 6868 patients (4033 boys and 2835 girls) ranging from 0 to 16 years old and divided the patients into five age groups: < 1 year (group I), 1–< 3 years (group II), 3–< 6 years (group III), and > 6 years (group IV). This study adhered to the ethical principles of the Declaration of Helsinki and was approved by the ethics committee of the Children’s Hospital of Soochow University (No.2019KS004).

### IM and atypical EBV infection definitions

The diagnostic criteria for EBV-IM are as follows [12]: (1) the presence of at least three of the following clinical manifestations: fever, tonsillopharyngitis, cervical lymphadenopathy, hepatomegaly, and splenomegaly; (2) immunoglobin (Ig) M and IgG to EBV viral capsid antigen (VCA-IgM)-positive and IgG to nuclear antigen (EBNA)-negative or VCA-IgM-negative and VCA-IgG-positive (low affinity); and (3) other infections, such as cytomegalovirus and human immunodeficiency virus, were ruled out. In this study, atypical EBV infection was defined as the onset of fever or elevated atypical lymphocytes in the peripheral blood without target organ damage [13].

### Laboratory testing

#### Plasma EBV-DNA polymerase chain reaction (PCR)

Peripheral blood was obtained from the admitted patients and immediately centrifuged for examination. First, 10 μL of plasma was mixed with 10 μL of DNA extract (Shengxiang Biotechnology Co., Ltd., Hunan, China). Next, 40 μL of the PCR mixture was added and centrifuged at 2000 rpm for 30 s. Real-time quantitative PCR was performed on a LightCycler 480II instrument (Roche, Basel, Switzerland) under the following conditions: 50 °C for 2 min, then 94 °C for 2 min, followed by 45 cycles of 94 °C for 5 s, 57 °C for 30 s, and 25 °C for 10 s. Critical, negative, positive, and four positive controls (10,^4^ 10,^5^ 10,^6^ and 10^7^ copies/mL, respectively) were set for each test. At the end of the reaction, the viral DNA loads were calculated by comparing the cycle threshold (Ct) of the specimens to the standard curve. EBV negativity was defined as the Ct value of the negative control, and EBV positivity was defined as a Ct value of ≤39 (DNA copy number > 400 copies/mL).

#### EBV-specific antibodies by indirect immunofluorescence

The anti-EBV-VCA IgG/IgM, anti-EBV-early antigen (EA) IgG, and anti-EBNA IgG indirect immunofluorescence (i.e., IIF) kits (Euroimmun, Lübeck, Germany) were used to measure the serum EBV-VCA, EA, and EBNA levels. All steps were performed following the manufacturer’s instructions. Antibody affinity was determined by comparing the fluorescence intensities of serum from children with and without urea treatment. A fluorescence intensity difference of ≥2 was considered low affinity, and a difference of < 2 was considered high affinity.

#### ADA and ALT

Peripheral blood was obtained from the admitted patients. Serum ALT and ADA levels were measured using lactate dehydrogenase assay (Beijing Strong Biotechnologies, Inc., Beijing, China) and peroxidase assay (Meikang Biotechnology Co., Ltd., Shanghai, China) kits, respectively. Both were detected using a Hitachi 7180 biomedical analyzer (Hitachi High-Tech Corp., Tokyo, Japan). Normal (reference) values for ALT and ADA were < 40 U/L and < 25 U/L, respectively.

### Statistical analyses

Data are presented as numbers (percentages) or medians (interquartile ranges). All statistical analyses were performed using IBM SPSS Statistics for Windows version 25 (IBM Corp., Armonk, NY, USA). Wilcoxon and Kruskal-Wallis tests were used for continuous variables with skewed distributions. Categorical variables were analyzed using the chi-squared test. Univariate and multivariate logistic regression analyses were used to determine the odds ratios (ORs) with 95% confidence intervals (CIs). Spearman’s correlation analysis was used to determine the correlation between discrete variables. Statistical significance was set at *P* < 0.05.

## Results

### Patient characteristics

We included 6868 inpatients who underwent plasma EBV-DNA, ALT, and ADA assays. The average age was 4.3 (± 3.0) years, with a male-to-female ratio of 1:4. The plasma EBV DNA positivity rate was 7.0% (483/6868); 1877 (27.3%) and 1667 (24.3%) children had elevated ADA and ALT levels, respectively.

The inpatients were divided into four categories based on their discharge diagnosis: inflammatory disease (*n* = 3030), autoimmune disease (*n* = 1006), malignant disease (*n* = 1061), and other diseases (*n* = 1065). Other diseases include anemia, hemophilia, jaundice, gastrointestinal malformations, inherited metabolic diseases, immunodeficiency diseases, facial paralysis, epilepsy, skin rashes, cardiomyopathy, and congenital heart disease.

### General characteristics based on the ADA level

Overall, 4991 patients had a normal ADA level, and 1877 had an elevated level. Sex, age, the ALT level, plasma EBV DNA positivity rate, and the disease category significantly differed between the normal and elevated ADA groups (*P* < 0.05; Table 1). An elevated ADA level was most common in girls, those aged < 3 years, and those with inflammatory diseases, a high ALT level, and EBV positivity.

**Table 1:** Comparison of clinical characteristics in patients with normal and elevated ADA

Parameters	ADA normal group (*n* = 4991)	ADA elevated group (*n* = 1877)	*P*
Gender(male)	2981 (59.7)	1052 (56.0)	0.006
Age(years)			
≤ 1	600 (12.0)	314 (16.7)	< 0.001
1-3	1340 (26.9)	607 (32.3)	< 0.001
3-6	1487 (29.8)	485 (25.9)	0.001
>6	1564 (31.3)	471 (25.1)	< 0.001
ALT(U/L)	18.5 (11.8-36.5)	23.1 (14.6-45.8)	< 0.001
EBV-DNA positive rate	111 (2.2)	372 (19.8)	< 0.001
Disease			
Inflammatory disease	1829 (36.7)	1201 (64.0)	< 0.001
Malignant disease	1483 (29.7)	284 (15.1)	< 0.001
Autoimmune disease	754 (15.1)	252 (13.4)	0.079
Other diseases	925 (18.5)	140 (7.5)	< 0.001

The data are reported as median (interquartile range) or n (%). *Abbreviation*: *ADA* Adenosine deaminase, *ALT* Alanine aminotransferase, *EBV* Epstein-Barr virus

### Elevated ADA risk factors

Univariate regression analysis identified associations between an elevated ADA level and the ALT level, EBV infection, and the disease category (Table 2). Furthermore, multivariate regression analysis adjusted for sex and age indicated that the ALT level (adjusted OR [aOR] = 1.001, 95% CI: 1.001–1.002), EBV infection (aOR = 8.486, 95% CI: 6.753–10.663), inflammatory disease (aOR = 3.915, 95% CI: 3.198–4.794), autoimmune disease (aOR = 2.307, 95% CI: 1.823–2.920), and malignant disease (aOR = 1.381, 95% CI: 1.101–1.734) were risk factors for an elevated ADA level; of them, EBV infection was the strongest risk factor.

**Table 2:** Univariable and multivariable logistic regression models of factors associated with elevated ADA in hospitalized patients

Variable	Model 1		Model 2		Model 3	
	OR (95%CI)	*P*	OR (95%CI)	*P*	OR (95%CI)	*P*
ALT(U/L)	1.001 (1.000-1.002)	0.001	1.001 (1.001-1.002)	< 0.001	1.001 (1.001-1.002)	< 0.001
EBV-DNA	10.895 (8.746-13.572)	< 0.001	11.702 (9.368-14.617)	< 0.001	8.486 (6.753-10.663)	< 0.001
Diseases						
Inflammatory disease	4.339 (3.580-5.257)	< 0.001	4.402 (3.625-5.345)	< 0.001	3.915 (3.198-4.794)	< 0.001
Malignant disease	1.265 (1.017-1.572)	0.035	1.357 (1.088-1.694)	0.007	1.381 (1.101-1.734)	0.005
Autoimmune disease	2.202 (1.754-2.766)	< 0.001	2.155 (1.713-2.711)	< 0.001	2.307 (1.823-2.920)	< 0.001
Other diseases	1(reference)		1(reference)		1(reference)	

*Abbreviations*: *ADA* Adenosine deaminase, *ALT* Alanine aminotransferase, *OR* Odds ratio, *CI* Confidence interval, *EBV* Epstein-Barr virus. Model 1 was not adjusted. Model 2 was adjusted for age and gender. Model 3 was adjusted as Model 2 + ALT, EBV-DNA positive, diseases

### The ADA level in EBV-related diseases

Based on the discharge diagnosis, 483 children were plasma EBV-positive and diagnosed with a respiratory infection, IM, atypical EBV infection, malignant disease (e.g., hemophagocytic lymphohistiocytosis and tumors), and other diseases. Other diseases include Kawasaki disease, idiopathic thrombocytopenic purpura, epilepsy, stomatitis, and meningitis. Furthermore, 279 children had a normal ALT level, and 204 had an elevated level.

**Fig. 1:**
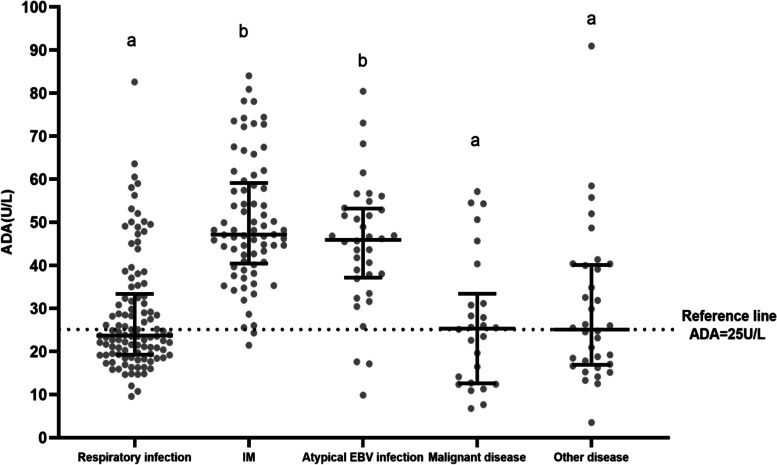
The value of ADA in patients with EBV-related diseases. *P < 0.05 (a versus b)

Of those with a normal ALT level, significantly more children with IM (97.3%) and atypical EBV infection (91.7%) had an elevated ADA level than those with a respiratory infection (45.8%), malignant disease (57.1%), or other diseases (51.4%) (*P* < 0.05; Table 3 and Fig. 1). Moreover, the ADA concentration was significantly higher in the IM and atypical EBV infection groups than in the other three groups (*P* < 0.05).

**Table 3:** The value of ADA in children with positive plasma EBV-DNA

Diseases				Normal ALT				Elevated ALT		
	*N* = 483	*n* = 279	Gender (male)	ADA [U/L,M(P_25_,P_75_)]	ADA↑ n(%)	*n* = 204	Gender (male)	ADA [U/L,M(P_25_,P_75_)]	ADA↑ n(%)	ALT [U/L,M(P_25_,P_75_)]
Respiratory infection	131	107	62 (57.9)	23.8 (19.3-35.0)a	49 (45.8)a	24	17 (70.8)	51.6 (41.2-60.1)ac	22 (91.7)a,b	57.9 (43.6-111.7)a
IM	161	73	46 (63.0)	47.2 (40.4-59.1)b	71 (97.3)b	88	42 (47.7)	65.9 (54.2-77.4)b	88 (100.0)b	77.9 (49.8-119.7)ab
Atypical EBV infection	96	36	23 (63.9)	45.9 (37.1-53.2)b	33 (91.7)b	60	33 (55.0)	55.4 (48.6-72.4)ab	56 (93.3)a,b	102.2 (74.2-162.8)b
Malignant disease	50	28	13 (46.4)	25.6 (13.0-44.3)a	16 (57.1)a	22	12 (54.5)	28.3 (12.9-47.5)c	12 (54.5)c	65.5 (51.7-110.7)ab
Other diseases	45	35	19 (54.3)	25.5 (17.0-40.3)a	18 (51.4)a	10	6 (60.0)	40.2 (20.7-71.1)abc	7 (70.0)a,c	65.3 (46.9-140.3)ab
*P*			0.559	< 0.001	< 0.001		0.367	< 0.001	< 0.001	< 0.001

The data are reported as median (interquartile range) or n (%). *Abbreviation*: *ADA* Adenosine deaminase, *ALT* Alanine aminotransferase, *EBV* Epstein-Barr virus, *IM* Infectious mononucleosis. *P* < 0.05 between a, b and c

**Fig. 2:**
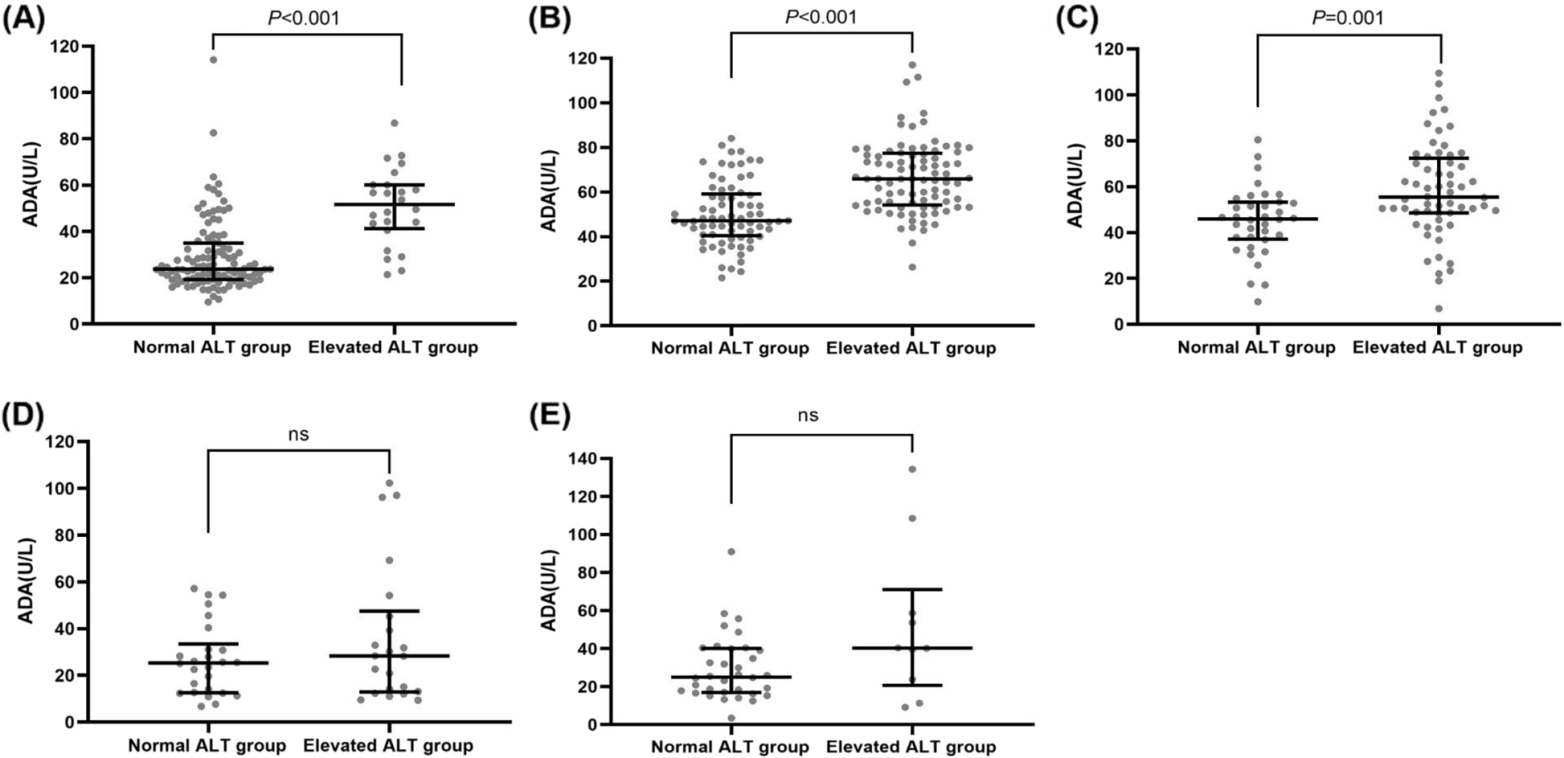
ADA in normal and elevated ALT groups from patients with positive plasma EBV-DNA. **A** The value of ADA in normal and elevated ALT groups from patients with respiratory infection. **B** The value of ADA in normal and elevated ALT groups from patients with IM. **C** The value of ADA in normal and elevated ALT groups from patients with atypical EBV infection. **D** The value of ADA in normal and elevated ALT groups patients with malignant disease. **E** The value of ADA in normal and elevated ALT groups from patients with other diseases

Finally, patients with IM, atypical EBV infection, and respiratory infection in the elevated ALT level group had higher ADA levels than those in the normal ALT level group (Fig. 2).

### Correlations between the ADA level and EB viral load in children with a normal ALT level

**Fig. 3:**
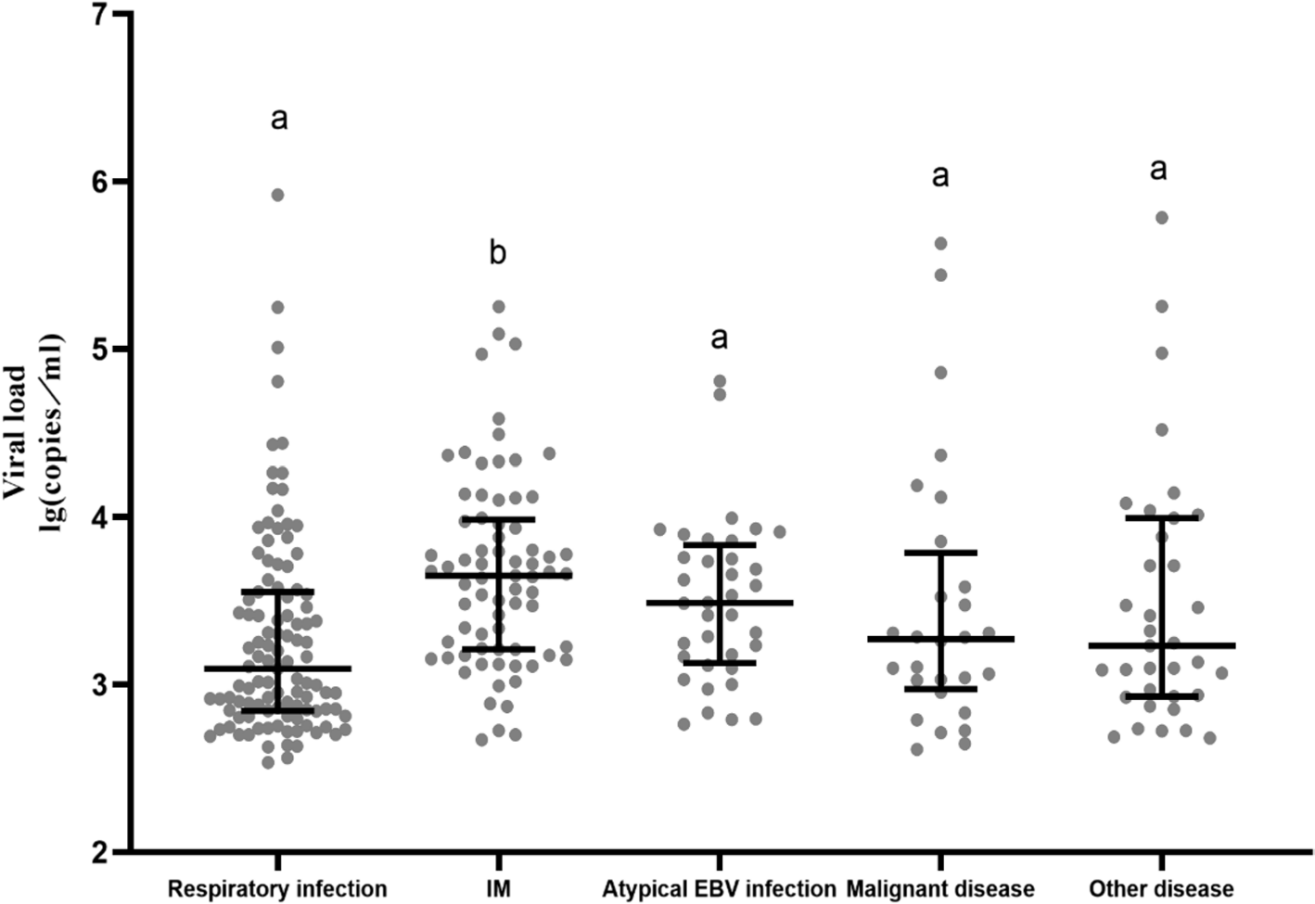
The viral loads of children with EBV-related diseases. * P < 0.05 (a versus b)

**Fig. 4:**
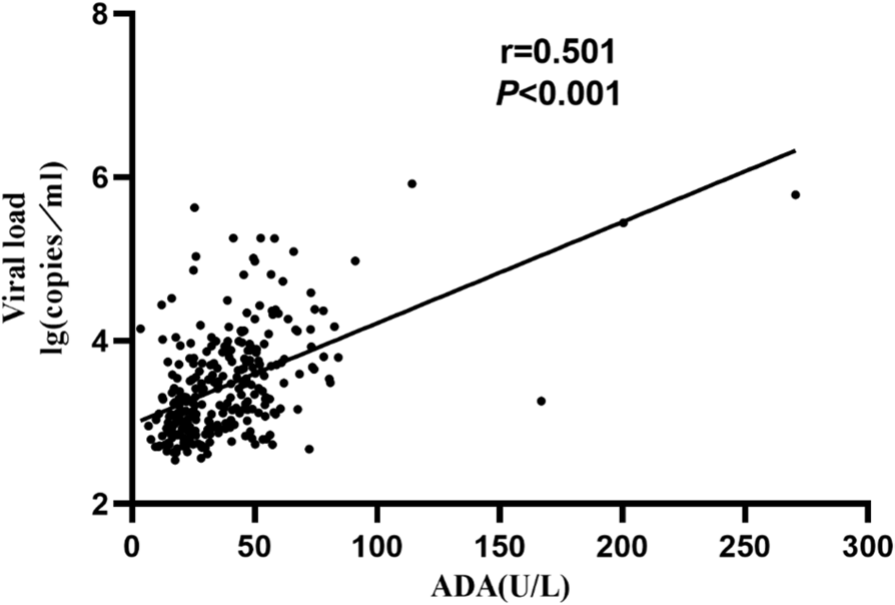
Correlation between EB virus load and ADA in patients with normal ALT

The EB viral load was significantly higher in the IM group than in the atypical EBV infection, respiratory infection, malignant disease, and other disease groups (Fig. 3). Furthermore, Spearman’s correlation analysis showed that the EB viral load positively correlated with the ADA level (r = 0.501, *P* < 0.05; Fig. 4).

## Discussion

ADA is an enzyme involved in purine metabolism with an important role in cellular immunity. Furthermore, it is primarily located in lymphoid tissues and is involved in cellular immune responses [14]. However, ADA is also present in hepatocytes, and when hepatocytes are damaged, the ADA level in the peripheral blood increases [15]. For example, the ADA level is notably elevated in *Mycobacterium tuberculosis* infection, autoimmune diseases, hepatitis, and malignant tumors [2–4, 16]. Thus, ADA’s expression and activity increase significantly during inflammation, and monocytes and macrophages are important sources of extracellular ADA [17]. ADA binds to dipeptidyl peptidase-4 (CD26), expressed in cluster of differentiation (CD)4^+^T lymphocyte helper and memory T cells to form a complex [18]. This complex alleviates the inhibitory effect on T lymphocyte proliferation and activation by reducing local adenosine concentrations. In addition, it promotes the differentiation of naive T lymphocytes into helper and memory T cells and increases the generation of CD4^+^, CD25^+^, and forkhead box P3 (i.e., Foxp3)^+^ regulatory T cells. At the immune synapse between dendritic cells and CD4+ T cells, the CD26-ADA complex acts as a co-stimulator to induce interleukin-6 production and increase interferon-γ and tumor necrosis factor-α secretion, enhancing the inflammatory response [19, 20]. These events indicate that ADA plays an important role in T-lymphocyte immunity. In this study, we used non-inflammatory, congenital, and metabolic diseases as references, finding that an elevated ADA level was the most common in inflammatory diseases, followed by autoimmune and malignant diseases. However, EBV infection is also strongly associated with the ADA level.

EBV infection activates the immune response mediated by cytotoxic T cells, which is closely related to various diseases, such as respiratory infections, IM, hemophagocytic lymphohistiocytosis, and malignant tumors [11]. Furthermore, the diversity of EBV infections is closely related to immune function [8]. Of the EBV-DNA-positive children, more than 90% of those with IM and atypical EBV infection had an elevated ADA level, suggesting that the ADA level is highly sensitive for diagnosing EBV-IM and atypical EBV infection if the specificity of ADA alone is relatively low. In contrast, only half of the children with EBV-related respiratory infections and malignant diseases had an elevated ADA level, likely because EBV infection may not be the primary cause of the disease or the EBV infection did not elicit a strong immune response [8, 21].

EBV DNA in plasma signals an active EBV infection [22]. After excluding the effects of ALT, we found that the plasma EBV load and the ADA level were higher in patients with IM, and the ADA level positively correlated with the EBV load. These results suggest that ADA is related to the immune response to EBV infection and lytic EBV infection. Previous in vivo studies determined that EBV-encoded RNA 2 (EBER-2), a small, non-translated transcript, helps protect the EBV from antiviral effects while the latent infection is being established [23]. The mechanism involves three complementarity regions between EBER-2 and human ADA messenger RNA, leading to inhibited ADA translation [24]. ADA removes excess extracellular adenosine, a well-established monocyte, cytotoxic T lymphocyte, and neutrophil inhibitor [25]. Thus, a high level of secreted adenosine causes a downregulation of the immune response against EBV-infected cells, potentially promoting latent infection. We hypothesize that this was why our study did not find significantly elevated ADA levels in EBV-related malignancies. Relatively low ADA levels have also been detected in the EBV-producing cell lines B95-8 and P3HR-1 [26].

Some studies have indicated that the plasma EBV copy number could be a disease severity and prognostic biomarker for EBV-related diseases [27]. For example, Chen et al. [28] found an increased serum ADA level in patients with secondary haemophagocytic lymphohistiocytosis. However, in this study, the ADA level was normal in those with EBV-related malignant diseases, which may be related to ADA’s involvement in metabolic and immune signaling pathways.

This study has some limitations. First, this is a retrospective study. Second, further prospective work on the mechanism of action of ADA in EBV infection-related diseases need to be carried out.

## Conclusion

This large, retrospective study is the first to explore the correlation between ADA, EBV-related diseases, and the EB load, identifying differences in the ADA levels among EBV-related diseases and a positive correlation between the ADA level and the EB viral load; these results may help clinicians detect these diseases earlier based on the plasma ADA concentration. According to previous literature reports, the diversity exhibited by ADA among EBV-related diseases may be related to its involvement in T-lymphocyte-mediated immune responses, EBV immune escape in infected cells, and hepatocyte destruction. That will provide new ideas for further exploring the mechanism of ADA in EBV-related diseases.

## Data Availability

The data that support the findings of this study are available from the corresponding author upon reasonable request (2231365607@qq.com).

## Abbreviations

ADA: Adenosine deaminase
EBV: Epstein–Barr virus
ALT: Alanine aminotransferase
OR: Odds ratio
CI: Confidence interval
IM: Infectious mononucleosis
CTL: Cytotoxic T-lymphocytes
VCA: Viral capsid antigen
NA: Nuclear antigen
CMV: Cytomegalovirus
HIV: Human immunodeficiency virus

## References

[1] Antonioli L, Colucci R, La Motta C, Tuccori M, Awwad O, Da Settimo F (2012). Adenosine deaminase in the modulation of immune system and its potential as a novel target for treatment of inflammatory disorders. Curr Drug Targets.

[2] Ghaderi B, Amini S, Maroofi F, Jalali C, Javanmardi M, Roshani D (2016). Adenosine deaminase activity in chronic lymphocytic leukemia and healthy subjects. Iran. J Cancer Prev.

[3] Naval-Macabuhay I, Casanova V, Navarro G, García F, León A, Miralles L (2016). Adenosine deaminase regulates Treg expression in autologous T cell-dendritic cell cocultures from patients infected with HIV-1. J Leukoc Biol.

[4] Yordanova M, Gerova D, Atanassova A, Galunska B. Adenosine deaminase as a useful biomarker for diagnosis and monitoring of inflammatory bowel disease. Clin Lab. 2020;66. 10.7754/Clin.Lab.2019.191124. 10.7754/Clin.Lab.2019.191124 32658411

[5] Kaya S, Cetin ES, Aridogan BC, Arikan S, Demirci M (2007). Adenosine deaminase activity in serum of patients with hepatitis -- a useful tool in monitoring clinical status. J Microbiol Immunol Infect.

[6] Mejer J, Nygaard P, Cohn J, Gadeberg O, Faber V (1984). Adenosine deaminase, purine nucleoside phosphorylase and 5′-nucleotidase activities in infectious mononucleosis. Adv Exp Med Biol.

[7] Cohen JI (2000). Epstein-Barr virus infection. N Engl J Med.

[8] Nowalk A, Green M. Epstein-Barr virus. Microbiol Spectr. 2016;4. 10.1128/microbiolspec.DMIH2-0011-2015. 10.1128/microbiolspec.DMIH2-0011-2015 27337443

[9] Macsween KF, Crawford DH (2003). Epstein-Barr virus-recent advances. Lancet Infect Dis.

[10] Balfour HH, Verghese P (2013). Primary Epstein-Barr virus infection: impact of age at acquisition, coinfection, and viral load. J Infect Dis.

[11] Fugl A, Andersen CL (2019). Epstein-Barr virus and its association with disease - a review of relevance to general practice. BMC Fam Pract.

[12] Shi J, Ma W, Li W (2020). Epidemiologic features of children with Epstein-Barr virus associated diseases in Hangzhou, China. J Med Virol.

[13] Shi T, Huang L, Chen Z, Tian J (2021). Characteristics of primary Epstein-Barr virus infection disease spectrum and its reactivation in children, in Suzhou, China. J Med Virol.

[14] Van der Weyden MB, Kelley WN (1976). Human adenosine deaminase. Distribution and properties. J Biol Chem.

[15] Fernández E, Rodrigo L, Riestra S, Carcía S, Gutiérrez F, Ocio G (2000). Adenosine deaminase isoenzymes and neopterin in liver cirrhosis. J Clin Gastroenterol.

[16] Wen P, Wei M, Han C, He Y, Wang MS (2019). Risk factors for tuberculous empyema in pleural tuberculosis patients [Sci. Rep.]. Sci Rep.

[17] Fischer D, Van der Weyden MB, Snyderman R, Kelley WN (1976). A role for adenosine deaminase in human monocyte maturation. J Clin Invest.

[18] Casrouge A, Sauer AV, Barreira da Silva R, Tejera-Alhambra M, Sánchez-Ramón S, ICAReB (2018). Lymphocytes are a major source of circulating soluble dipeptidyl peptidase 4. Clin Exp Immunol.

[19] Martinez-Navio JM, Casanova V, Pacheco R, Naval-Macabuhay I, Climent N, Garcia F (2011). Adenosine deaminase potentiates the generation of effector, memory, and regulatory CD4+ T cells. J Leukoc Biol.

[20] Yu DM, Slaitini L, Gysbers V, Riekhoff AG, Kähne T, Knott HM (2011). Soluble CD26 / dipeptidyl peptidase IV enhances human lymphocyte proliferation in vitro independent of dipeptidyl peptidase enzyme activity and adenosine deaminase binding. Scand J Immunol.

[21] Huang Y, Wei C, Zheng K, Zhao D (2013). The impact of serological features in Chinese children with primary or past Epstein-Barr virus infections. Virol J.

[22] Kanakry JA, Hegde AM, Durand CM, Massie AB, Greer AE, Ambinder RF (2016). The clinical significance of EBV DNA in the plasma and peripheral blood mononuclear cells of patients with or without EBV diseases. Blood..

[23] Wang Y, Guo Z, Shu Y, Zhou H, Wang H, Zhang W (2017). BART miRNAs: an unimaginable force in the development of nasopharyngeal carcinoma. Eur J Cancer Prev.

[24] Gerlitz G, Elroy-Stein O (1998). Does EBV RNA modulate ADA mRNA translation?. Leukemia..

[25] Fredholm BB, Johansson S, Wang YQ (2011). Adenosine and the regulation of metabolism and body temperature. Adv Pharmacol.

[26] Margalith M, Manor D, Cohen T (1983). Cytotoxicity of arabinofuranosyladenine and erythro-9-(2-hydroxy-3-nonyl) adenine to Epstein-Barr virus producer and nonproducer lymphoma cells in culture. Cancer Biochem Biophys.

[27] Kimura H, Kwong YL (2019). EBV viral loads in diagnosis, monitoring, and response assessment. Front Oncol.

[28] Chen W, Zhang S, Zhang W, Yang X, Xu J, Qiu H (2015). Elevated serum adenosine deaminase levels in secondary hemophagocytic lymphohistiocytosis. Int J Lab Hematol.

